# Possible modulation of FAS and PTP-1B signaling in ameliorative potential of *Bombax ceiba* against high fat diet induced obesity

**DOI:** 10.1186/1472-6882-13-281

**Published:** 2013-10-25

**Authors:** Paras Gupta, Rohit Goyal, Yamini Chauhan, Pyare Lal Sharma

**Affiliations:** 1 Department of Pharmacology, ISF College of Pharmacy, Moga 142 001, India; 2 School of Pharmaceutical Sciences, Shoolini University, Solan, HP 173212, India

**Keywords:** Obesity, *Bombax ceiba*, Free fatty acid, Protein tyrosine phosphatase-1B, Fatty acid synthase

## Abstract

**Background:**

*Bombax ceiba* Linn., commonly called as Semal, is used in various gastro-intestinal disturbances. It contains Lupeol which inhibits PTP-1B, adipogenesis, TG synthesis and accumulation of lipids in adipocytes and adipokines whereas the flavonoids isolated from *B. ceiba* has FAS inhibitory activity. The present study was aimed to investigate ameliorative potential of *Bombax ceiba* to experimental obesity in Wistar rats, and its possible mechanism of action.

**Methods:**

Male Wistar albino rats weighing 180-220 g were employed in present study. Experimental obesity was induced by feeding high fat diet for 10 weeks. Methanolic extract of *B. ceiba* extract 100, 200 and 400 mg/kg and Gemfibrozil 50 mg/kg as standard drug were given orally from 7^th^ to 10^th^ week.

**Results:**

Induction with HFD for 10 weeks caused significant (p < 0.05) increase in % body wt, BMI, LEE indices; serum glucose, triglyceride, LDL, VLDL, cholesterol, free fatty acid, ALT, AST; tissue TBARS, nitrate/nitrite levels; different fat pads and relative liver weight; and significant decrease in food intake (g and kcal), serum HDL and tissue glutathione levels in HFD control rats. Treatment with *B. ceiba* extract and Gemfibrozil significantly attenuated these HFD induced changes, as compared to HFD control. The effect of *B. ceiba* 200 and 400 mg/kg was more pronounced in comparison to Gemfibrozil.

**Conclusion:**

On the basis of results obtained, it may be concluded that the methanolic extract of stem bark of *Bombax ceiba* has significant ameliorative potential against HFD induced obesity in rats, possibly through modulation of FAS and PTP-1B signaling due to the presence of flavonoids and lupeol.

## Background

Obesity is one of the leading causes of death worldwide [1] and characterized by excess body fat accumulation [2]. It is a chronic disorder with complex interaction between genetic and environmental factors and occurs due to increased intake of high fat and energy food with decreased energy expenditure. Endogenous involvement of leptin, insulin, ghrelin, CCK (cholecystokinin), NPY (neuropeptide Y), GLP-1 (glycogen like peptide 1) and PTP-1B (protein tyrosine phosphate-1B) signaling has potential effect on food intake and energy expenditure [3]. The stimulation of PTP-1B also modulates insulin, leptin and integrin signaling, and thereby stimulates fatty acid synthase (FAS) activity and results obesity [4,5]. According to Ayurveda, vitiations in three body humours i.e. tridoshas (vata, pitta and kapha) is characterized by altered body functions like metabolism, digestion, appetite and thus precipitates obesity [6]. The clinical claims revealed that the obesity is caused due to genetic predisposition and improper life style.

The traditional system of medicine has incorporated use of medicinal plant drugs or formulation for the prevention of obesity. *Bombax ceiba* Linn., belongs to family Malvaceae, commonly called as semal, shimbal (in hindi) and red silk cotton tree (in english) [7]. The stem bark is reported to contain lupeol, shamimicin, flavonoids, glycoside, sterol: β-sitosterol, terpenoids, napthol, hemigossylic acid and lactone-7-methyl ether [8,9]. Shamimin (C-flavonol glucoside) isolated from *B. ceiba* methanolic extract showed significant hypotensive and hypoglycaemic properties and was found to be safe in rodents [9]. Mangiferin obtained from methanolic extract of *B. ceiba* leaves demonstrated strong antioxidant and hepatoprotective activities [10]. Ethno-pharmacologically, it is used to treat diarrhea, dysentery, digestive disturbances, diabetes [11] and improves digestion (Jain, 1996). In ayurveda, *B. ceiba* is generally recommended to use in vitiated conditions of vata, pitta and kapha and removes pitta and kappa [12]. It has a potent free radical scavenging [13], anti-inflammatory and hepatoprotective activities [8,14]. The flavonoids isolated from *B. ceiba* have a potent FAS inhibitory activity [15]. Lupeol, found in *B. ceiba* inhibits PTP-1B, adipogenesis, TG synthesis and accumulation of lipids in adipocytes and adipokines [16].

On the basis of literature available, we hypothesized to investigate the possible anti-obesity potential of *Bombax ceiba* in high fat diet-induced experimental obesity, possibly due to the involvement of FAS and PTP-1B signaling in present study.

## Methods

### Collection, authentication and extraction of plant material

*Bombax ceiba* Linn. stem bark was collected from Gwalior, MP, India, authentified from NISCAR, New Delhi under consultation with Dr. H.B. Singh, Director and a voucher specimen of plant drug sample was deposited in institutional herbarium (NISCAIR/RHMD/Consult/-20011-12/1758/58). Stem bark was shade dried, coarsely powdered and stored in air tight container till further use. The literature revealed that the phytoconstituents isolated from methanolic extract of *B. ceiba* have potent biological efficacies. Therefore, plant drug extraction was made with soxhlet extractor using methanol as solvent.

### Phytochemical screening

The qualitative phytochemical screening of *B. ceiba* extract was carried out for the presence of phytoconstituents like steroids, terpenoids, anthraquinone glycosides, C-glycosides, cardiac glycosides, flavonoids, tannins, phenolic and carbohydrates [17].

### Acute toxicity study

Acute toxicity study of *B. ceiba* extract was conducted as per the Organization for Economic Co-operation and Development (OECD) 423 guidelines: acute toxicity class method [18] using Wistar albino rats (n = 3). *B. ceiba* extract (methanolic) was given orally in doses: 50, 100, 300, 1000 and 2000 mg/kg; suspended in 0.5% CMC solution and the animals were observed for physiological (body wt, urination, pellet expulsion and salivation), behavioral (irritability, corneal reflex, catatonia, locomotion, convulsion and tremor), biochemical (serum ALT, AST and glucose) and toxic manifestations and even mortality, if any, up to 14 days.

### Chemicals and reagents

Casein from Modern Dairy, New Karnal, India; cholesterol from Thomas Baker; and Gemfibrozil from Pfizer, USA were purchased. The biochemical enzymatic kits were purchased from Coral Diagnostics Ltd., Mumbai, India. All other chemicals/reagents used were of analytical grade and were freshly prepared before use.

### Animals

Male, Wistar albino rats, weighing 180-220 g were employed in present study. They were fed on standard chow diet (Ashirwad Industries Private Ltd., Ropar, Punjab, India) and water *ad libitum*; and maintained at 12-12 h light/dark cycles, temperature 25 ± 2°C and relative humidity 55 ± 5%. The experimental protocol was duly been approved by Institutional Animal Ethics Committee (IAEC) and the experimentations were conducted under the guidelines from Committee for the purpose of Control and Supervision of Experiments on Animals (CPCSEA).

### High fat diet-induced obesity

Experimental obesity was developed by feeding high fat diet (Powdered Normal Chow, 365 g; lard, 310 g; casein, 250 g; cholesterol, 10 g; vitamin mix and mineral mix, 60 g; dl-methionine, 03 g; yeast powder, 01 g; and NaCl, 01 g were mixed to prepare 1.0 kg of HFD) [19], to rats for 10 weeks. The High fat diet contains 5.33 kcal/g while the normal chow contains 3.80 kcal/g.

### Experimental protocol

All animals were divided into different groups each comprising six animals (n = 6). The groups were 1). Normal control receiving vehicle only; 2). HFD control receiving high fat diet for 10 weeks; 3). Gemfibrozil-50 receiving gemfibrozil 50 mg/kg as standard; 4), 5), 6) B.C. 100 or 200 or 400 receiving *B. ceiba* extract 100, 200 and 400 mg/kg respectively. Administration of vehicle, standard and the plant extracts were done orally from 7^th^ week to 10 week by suspending in 0.5% CMC solution as vehicle. After completion of experimental protocol, pahramcological assessments shall be carried out. Animals were anaesthetized, blood was collected from retro orbital plexuses, centrifuged and serum separated for biochemical estimations. Animals were sacrificed, and liver and different fat depots were surgically dissected out. 10% liver homogenate was prepared in 0.1 M Tris buffer (pH = 7.4) or phosphate buffer (0.1 M, pH = 7.4) (for glutathione only) for tissue biochemical estimations.

### Pharmacological assessment

#### Assessment of anthropometric parameters

The body mass index (BMI) [20] and Lee index [21] were assessed as an index of obesity. Body weight and food intake (g and kcal) were assessed weekly. Weight of liver and different fat depots: epididymal, retroperitoneal and mesenteric fat depots, total weights were also estimated [22].

### Assessment of serum biochemical parameters

The estimation of serum glucose [23]; total cholesterol [24]; high density lipoprotein (HDL) [24], low density lipoprotein LDL-[25]; very low density lipoprotein VLDL [25]; triglycerides [26]; and ALT, AST [27] were carried out spectrophotometrically using biochemical enzymatic kits (Coral Diagnostics Ltd., Mumbai, India).

### Serum fatty acid estimation by gas chromatography (GC)

The fatty acid estimation by gas chromatography was done by modified method of Christie [28]. The fatty acid was converted to fatty acid ester. For esterification, 300 μl of serum was made upto 3 ml with methanol then 5 drops of concentrated HCl were added. Final step for esterification was to reflux for 6 hrs. After esterification, to 500 μl of sample, added 300 μl of distilled water and toluene, shaked well and centrifuged for 5 minutes. 1 μl of organic solvent was injected into packed column of GC. Standard curve of different FAs was determined using different concentrations: 100-1000 μM of oleic and palmitic acids. The conditions for GC were: 40-160 (10°C/min), 160-230 (5°C/min), 230-240 (1°C/min), and stable for 10 min at 240C.

### Assessment of tissue biochemical parameters

The tissues were homogenized in 0.1 M tris buffer (pH 7.4) or 0.1 M phosphate buffer (for glutathione only) using teflon-glass homogenizer. The 10% liver homogenates were subjected for following tissue biochemical estimations:

### Lipid per oxidation (TBARS)

Lipid per-oxidation was determined by measuring the amounts of malondialdehyde (MDA) produced primarily or thiobarbituric acid reactive substances (TBARS), according to the modified method of Ohkawa *et al*[29]. Briefly, 0.2 ml of tissue homogenate, 0.2 ml of 8.1% sodium dodecyl sulphate or sodium lauryl sulphate (SDS/SLS), 1.5 ml of 20% acetic acid and 1.5 ml of 8% TBA were added. The volume of the mixture was made up to 4 ml with distilled water and then heated at 95°C on a water bath for 60 min using glass balls as condenser. After incubation the tubes were cooled to room temperature the upper organic layer was taken and its OD read at 532 nm against an appropriate blank without the sample. The levels of lipid peroxides were expressed as nmoles of thiobarbituric acid reactive sub-stances (TBARS) nmol/mg of liver wt.

### Reduced glutathione (GSH) [30]

The homogenate was added with equal volume of 20% trichloroacetic acid (TCA) containing 1 mM EDTA to precipitate the tissue proteins. The mixture was allowed to stand for 5 min prior to centrifugation for 10 min at 200 rpm. The supernatant (200 μl) was then transferred to a new set of test tubes and added 1.8 ml of the Ellman’s reagent (5, 5′-dithio *bis-*2-nitrobenzoic acid) (0.1 mM) was prepared in 0.3 M phosphate buffer with 1% of sodium citrate solution). Then all the test tubes make upto the volume of 2 ml. After completion of the total reaction, the absorbance of the solutions was estimated at 412 nm against blank. The level of GSH was expressed as μmol/mg of liver wt.

### Nitrite/nitrate level using Greiss reagent [31]

Tissue nitrite/nitrate was estimated using Greiss reagent which served as an indicator of nitric oxide production. An amount of 100 μl Greiss reagent (1:1 solution of 1% sulphanilamide in 5% phosphoric acid and 0.1% napthaylamine diamine dihydrochloric acid in water) was added to 100 μl of supernatant and absorbance was measured at 542 nm. Nitrite level was expressed as μmol/ mg of liver wt.

### Histopathological study

The liver and fat depots were excised out, washed in ice-cold saline, blotted dry and preserved in 10% formalin solution. The histopathological study was done using hematoxylin and eosin stains and observed under microscope to assess changes in liver tissue and size of fat depots [32,33].

### Statistical analysis

The results were expressed as mean ± standard deviation (SD) analyzed by one-way and two-way analysis of variance (ANOVA) followed by Bonferroni’s multiple comparison test as *post hoc* analysis. p < 0.05 was considered statistically significant.

## Results

### Extraction of plant materials

The yield of methanolic extract of *B. ceiba* Linn stem bark was found to be 4.69% w/w. Phytochemical screening of methanolic extract showed the presence of carbohydrate, alkaloids, C-glycosides, cardiac glycosides, saponins glycoside, tannins, terpenoids, steroids and flavonoids constituents.

### Acute toxicity study

*B. ceiba* extract at the doses of 50, 100, 300, 1000, 2000 mg/kg showed no significant physiological, behavioral and biochemical alterations and was found to be safe, and produced no toxic manifestation and mortality in rats. Hence, the plant extract in doses 100, 200 and 400 mg/kg, *p.o.* was selected for further pharmacological investigations.

### Effect of various pharmacological interventions on anthropometric parameters

**Figure 1:**
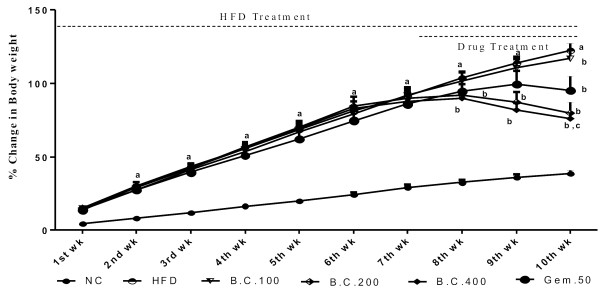
Effect of various pharmacological interventions on body weight; Results were expressed as mean ± SD; a = p < 0.05 vs NC, b = p < 0.05 vs HFD control, c = p < 0.05 vs Gem.50 on respective week.

**Figure 2:**
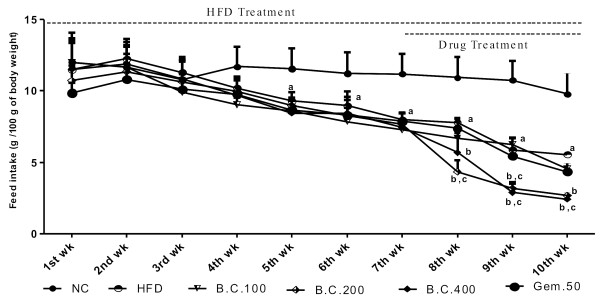
Effect of various pharmacological interventions on feed intake (g/100 g body wt); Results were expressed as mean ± SD; a = p < 0.05 vs NC, b = p < 0.05 vs HFD control, c = p < 0.05 vs Gem.50 on respective week.

**Figure 3:**
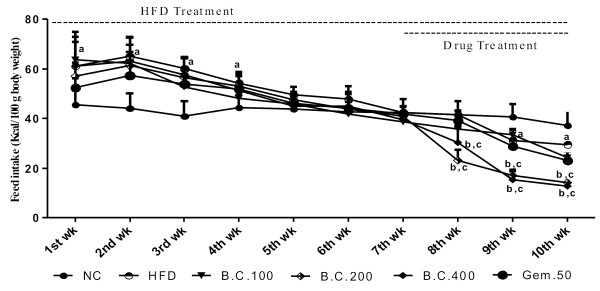
Effect of various pharmacological interventions on feed intake (Kcal); Results were expressed as mean ± SD; a = p < 0 .05 vs NC, b = p < 0.05 vs HFD control, c = p < 0.05 vs Gem.50 on respective week.

The high fat diet treatment for 10 weeks caused a significant (p < 0.05) increase in % body wt., BMI, Lee index, liver wt., wt. of fat depots and feed intake (kcal), and decrease in feed intake (g), as compared to normal control rats. Treatment with *B. ceiba* 100, 200 and 400 mg/kg and gemfibrozil 50 mg/kg produced significant (p < 0.05) attenuation of these changes caused due to chronic HFD, as compared to HFD control. The effect produced by *B. ceiba* 200 and 400 mg/kg was significant greater (p < 0.05), as compared to Gem.50, in all these parameters except % body wt. and feed intake (g and kcal) (Figures 1, 2 and 3) (Table 1).

**Table 1:** Effect of various pharmacological interventions on anthropometric parameters and weights of fat pads and liver

**Anthropometric parameters**						
**Group:**	**NC**	**HFD control**	**B.C. 100**	**B.C. 200**	**B.C. 400**	**Gem.50**
**BMI**	0.580 ± 0.0397	1.088 ± 0.028^a^	0.882 ± 0.025^b^	0.675 ± 0.071^b,c^	0.0.652 ± 0.054^b,c^	0.779 ± 0.028^b^
**Lee index**	303.51 ± 10.94	380.9 ± 4.07^a^	342.7 ± 2.79^b^	315.97 ± 15.98^b,c^	313.015 ± 15.033^b,c^	332.4 ± 5.63^b^
**Wt. of fat pads and liver wt. (g/100 g of body wt)**						
**Epididymal**	2.13 ± 0.42	4.583 ± 0.36^a^	3.05 ± 0.64^b^	1.3 ± 0.27^b,c^	1.28 ± 0.24^b,c^	2 ± 0.48^b^
**Mesenteric**	1.9 ± 0.11	4.85 ± 0.217^a^	3.95 ± 0.38^b^	2.15 ± 0.41^b,c^	2.17 ± 0.43^b,c^	3.1 ± 0.66^b^
**Retroperitoneal**	1.8 ± 0.43	5.28 ± 0.25^a^	3.93 ± 0.197^b^	2.13 ± 0.34^b,c^	2.03 ± 0.4^b,c^	3.23 ± 0.85^b^
**Total fat pads**	5.83 ± 0.612	14.72 ± 0.56^a^	10.93 ± 1.15^b^	5.58 ± 0.96^b,c^	5.48 ± 1.03^b,c^	8.3 ± 1.93^b^
**Liver wt.**	7.083 ± 0.231	13.30 ± 0.62^a^	12.98 ± 0.64	7.48 ± 0.42^b,c^	7.47 ± 0.52^b,c^	12.88 ± 0.96

Results were expressed as mean ± SD; ^a^p < 0.05 vs NC, ^b^p < 0.05 vs HFD control, ^c^p < 0.05 vs Gem.50.

### Effect of various pharmacological interventions on serum biochemical parameters

**Figure 4:**
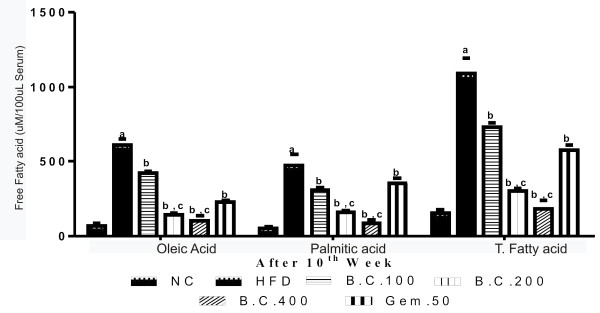
Effect of various pharmacological interventions on free fatty acids; Results were expressed as mean ± SD; a = p < 0.05 vs NC, b = p < 0.05 vs HFD control, c = p < 0.05 vs Gem.50.

The high fat diet treatment for 10 weeks caused a significant (p < 0.05) increase in serum glucose, triglyceride, LDL, VLDL, total cholesterol, free fatty acid, ALT, AST, and decrease in HDL levels, as compared to rats fed on standard chow diet. Treatment with *B. ceiba* extract in three doses, and Gem.50 significantly (p < 0.05) attenuated the increase in the levels of these serum markers, as compared to HFD control. The effect produced by *B. ceiba* 400 mg/kg was significantly greater than that of Gem-50, in all the parameters (Figure 4) (Table 2).

**Table 2:** Effect of various pharmacological interventions on serum and tissue biochemical parameters

**Serum biochemical parameters**						
**Group:**	**NC**	**HFD control**	**B.C. 100**	**B.C. 200**	**B.C. 400**	**Gem.50**
**Glucose (mg/dl)**	93.65 ± 13.46	202.25 ± 4.16^a^	177.77 ± 14.1^b^	95.8 ± 9.42^b,c^	90.8 ± 6.83^b,c^	100.3 ± 5.97^b^
**TG (mg/dl)**	78.9 ± 11.7	195.54 ± 10.63^a^	138.94 ± 5.8^b^	114.9 ± 11.75^b,c^	98.7 ± 7.83^b,c^	128.4 ± 15.3^b^
**TC (mg/dl)**	57.9 ± 4.8	343.42 ± 15.54^a^	169.889 ± 9.81^b^	95 ± 6.1^b,c^	93.12 ± 7.9^b,c^	94.93 ± 7.3^b^
**LDL (mg/dl)**	8.99 ± 5.42	289.76 ± 15.2^a^	120.83 ± 8.95^b^	26.45 ± 5.65^b,c^	13 ± 4.36^b,c^	15.1 ± 6.9^b^
**VLDL (mg/dl)**	15.78 ± 2.3	39.11 ± 2.12^a^	27.79 ± 1.17^b^	22.98 ± 2.4^b,c^	19.74 ± 1.6^b,c^	25.7 ± 3.1^b^
**HDL (mg/dl)**	33.13 ± 5.5	14.55 ± 1.9^a^	21.28 ± 5.84^b^	45.57 ± 8.4^b,c^	60.37 ± 3.1^b,c^	54.14 ± 5.1^b^
**ALT (IU/L)**	50.75 ± 6.6	153.26 ± 7.35^a^	94.28 ± 5.65^b^	67.9 ± 4.9^b,c^	50.8 ± 4.9^b,c^	71.8 ± 4.64^b^
**AST (IU/L)**	66.4 ± 6.7	160.83 ± 6.56^a^	107.86 ± 9.6^b^	65.38 ± 7.1^b,c^	50.25 ± 5.1^b,c^	87.5 ± 12.4^b^
**Tissue biochemical parameters**						
**TBARS**	0.6 ± 0.31	4.93 ± 0.64^a^	3.9 ± 0.77^b^	1.83 ± 0.7^b,c^	1.53 ± 0.7^b,c^	4.03 ± 0.71^b^
**Nitrite/nitrate**	0.74 ± 0.4	2.6 ± 0.1^a^	1.63 ± 0.04^b^	0.98 ± 0.1^b,c^	0.89 ± 0.69^b,c^	1.61 ± 0.02^b^
**Glutathione**	2.05 ± 0.25	0.19 ± 0.8^a^	0.42 ± 0.07^b^	1.8 ± 0.21^b,c^	1.99 ± 0.16^b,c^	0.55 ± 0.11^b^

Results were expressed as mean ± SD; ^a^p < 0.05 vs NC, ^b^p < 0.05 vs HFD control, ^c^p < 0.05 vs Gem.50.

### Effect of various pharmacological interventions on tissue biochemical markers

HFD treatment for 10 weeks in HFD control rats caused significant (p < 0.05) increase in tissue TBARS, nitrate/nitrite, and decrease in glutathione levels, as compared to rats fed on standard chow diet. Treatment with *B. ceiba* extract 100, 200 and 400 mg/kg and Gem.50 produced significant (p < 0.05) attenuation of these toxic changes produced by HFD in the dose dependent manner, as compared to HFD control rats. The effect produced by the plant extract 200 and 400 mg/kg was significantly greater (p < 0.05) than that produced by Gem.50 in all the parameters (Table 2).

### Effect of various pharmacological interventions on histology of liver and adipose tissue

**Figure 5:**
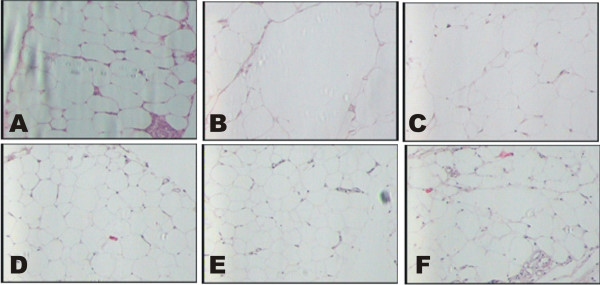
Effect of various pharmacological interventions on histology of adipose tissues; (A) NC, (B) HFD, (C) B.C. 100, (D) B.C. 200, (E) B.C. 400 and (F) Gem.50.

**Figure 6:**
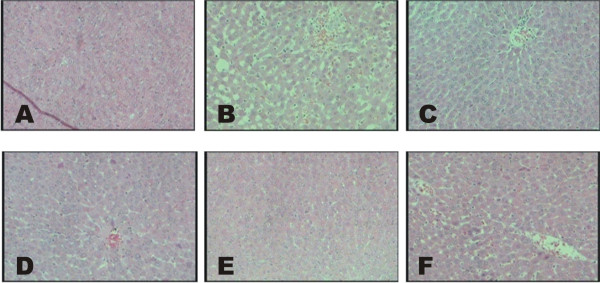
Effect of various pharmacological interventions on histology of liver tissue; (A) NC, (B) HFD, (C) B.C. 100, (D) B.C. 200, (E) B.C. 400 and (F) Gem.50.

High fat diet treatment for 10 weeks produced significant changes in hepatic tissue architecture such as micro & macro vascular steatosis, increased fatty infiltration, inflammation (over activation of kupffer cells), sinusoidal dilation, degeneration of central vein and vacuolization, as compared to normal liver histology. Treatment with *B. ceiba* 200 and 400 mg/kg significantly attenuated these effects of HFD, as compared to HFD control. Moreover, HFD treatment for 10 weeks produced significant increase in size of adipocytes: epididymal, retroperitoneal and mesenteric fat depots; as compared to rats fed on standard chow diet. Treatment with *B. ceiba* extract 200 and 400 mg/kg and Gem.50 significantly (p < 0.05) decreased the size of adipocytes, as compared to HFD control (Figures 5 and 6).

## Discussion

The present study demonstrated the anti-obesity effect of methanolic extract of *Bombax ceiba* Linn. against high fat diet-induced obesity in Wistar rats. Gemfibrozil is a lipid lowering agent, involved in metabolism of carbohydrates and fats, as well as adipose tissue differentiation and thereby prevents HFD induced obesity in rodents [34], and thus was used as a standard in present study. The experimental reports revealed that the methanolic or hydro-alcoholic extract has marked affinity for the active phytoconstituents of *B. ceiba.* Hence, the methanolic extract was prepared using soxhlet extractor. Characterization of *B. ceiba* extract was done in terms of phytochemical screening which signifies the presence of alkaloids, glycosides (cardiac-, saponin- glycosides), tannins, terpenoids, steroids and flavonoids in extract. The plant extract may be standardized for the presence of active phytochemical leads through modern analytical tools: HPLC or HPTLC in future endeavors. The acute toxicity study as per OECD guidelines derives the safe use of medicinal agent and its effects on physiological processes inside the body. Safety assessment is preferred to start with the assessment of biological effects of any bioactive agent. In acute toxicity study, the *B. ceiba* extract was found to be safe and did not reveal any biological defect and mortality in rodents.

High fat diet induced obesity is a commonly used model for experimental obesity and closely resembles with the symptoms of obesity in humans. HFD induced obesity is characterized by dyslipidemia, hyperglycemia and insulin resistance, increased fat accumulation, impaired glucose metabolism, distinctive visceral adiposity, hyperinsulinemia and hepatic steatosis in rodents [35,36]. In present study, high fat diet treatment for 10 weeks produced experimental obesity as evidenced by increased body weight, feed intake (Kcal), wt. of all fat depots, body mass index, Lee index, and decreased feed intake (g). Treatment with different doses of *B. ceiba* extract and standard drug (gemfibrozil) caused significant attenuation in these changes produced by HFD treatment. This effect may be due to the prevention of pathological mechanisms responsible for excessive fat accumulation, dyslipidemia and weight gain, possibly by increasing leptin sensitivity, providing anorexic effect, and increasing energy expenditure.

Lipogenesis up-regulation in HFD induced experimental obesity leads to elevated serum lipids [37] and decreased HDL levels in obese rats [36]. Further, it also produces hyperglycemia. The Free fatty acid level is reported to be increased during HFD induction [38]. It further caused liver damage and the increased level of hepatic serum markers like ALT and AST [39]. In present study, these serum markers have been modulated with chronic induction of HFD for 10 weeks as the markers of hyperlipidemia, dyslipidemia, hyperglycemia, and liver toxicity. On treatment with three consecutive doses of *B. ceiba* extract and gemfibrozil significantly reversed the effects of HFD treatment on these serum parameters. This may be due to the inactivation of acetyl-coA carboxylase (ACC), as a result of AMPK activation that mediates thermogenesis and FAS inhibition [15], which further inhibit the proximal and rate limiting steps of fatty acid oxidation [40]. This may derive the efficacy of *B. ceiba*’s to reduce circulating lipids, free fatty acids and thereby prevent fatty liver [41].

The increased fatty acid levels in chronically HFD fed rats is a characteristic marker caused due to the dysregulation of insulin and leptin signaling and stimulation of PTP-1B. These processes may be arrested by the administration of *B. ceiba* extract in rats and hence corrected the pharmacological interventions underlying the HFD induced obesity, as evidenced by the concentration of fatty acid content in biological fluid. These observations may be supported by the literatures revealing the efficacy of flavonoids isolated from *B. ceiba* extract having FAS inhibitory activity [15]. Moreover, the Lupeol, a triterpenoid, is a major constituent of stem bark of *B. ceiba* has inhibitory effect on PTP-1B, and thereby prevents TG synthesis and accumulation of lipids in adipocytes [16]. On the basis of the observations, it may be hypothesized that there is a possible modulation of FAS and PTP-1B signaling in protective effect of *B. ceiba* extract against HFD induced obesity and this may be due to the potency and efficacy of reported active phytoconstituents present in it.

Oxidative stress is greatly increased on the treatment with high fat diet in the form of enhanced lipid peroxidation reactions and depletion of tissue antioxidant like GSH; and higher nitrostative stress, in men [39]. In present study, increased TBARS and nitrite/nitrate, and decreased GSH levels confirm the role of oxidative and nitrostative stresses. Treatment with *B. ceiba* extract 100, 200 and 400 mg/kg significantly attenuated these HFD induced oxidative/nitrosative stress, and this effect was more pronounced, in comparison to standard drug: gemfibrozil. Moreover, another report documented the free radical scavenging property of *B. ceiba*[13]. Therefore, the anti-oxidative efficacy of *B. ceiba* may contribute for the amelioration of experimental obesity and hepatic insufficiencies in rats. In most of the pharmacological assessments, the biological efficacy of *B. ceiba* extract was more pronounced as compared to the standard drug: Gemfibrozil. These findings may infer that the methanolic extract of *B. ceiba* contain active phytoconstituents in higher concentration or acting synergistically to attain potent biological efficacy which was comparable to that of gemfibrozil. The future studies may lend support for the biological potency of isolated active phytoconstituentss like Lupeol, Shamimin etc of *B. ceiba* in sub-maximal therapeutic dosage and their comparative safe evaluation with reported lipid lowering agent. The findings of present investigation signifies the use of low dose of active phytoconstituent isolated from *B. ceiba* as much effective and may be devoid of any toxic effect on chronic administration in comparison to the reported lipid lowering agent.

Hepatic steatosis is a common consequence of obesity, and its prevalence [39] has been further characterized with hepatic fat accumulation and increase in size and wt of adipose masses in the body [42]. By increased liver steatosis, fatty infiltration, inflammation through Kupffer cell activation and size of adipose tissues epididymal, peritoneal, mesenteric fat depots and histological characteristics in present study as also observed in histological imagining. Various doses of *B. ceiba* and standard drug significantly reversed the effect of HFD on liver adipose tissues and liver steatosis, liver weight and adipose tissue size was increased during HFD treatment. Moreover extent of tissue architecture damage due to chronic HFD treatment was prevented by *B. ceiba* ext. effectively.

Therefore, in present study, the observed decrease in free fatty acid level may be due to the inhibition of FAS activity and TG synthesis, and PTP-1B activity. The antioxidant effect may also contribute to the anti-obesity potential of *Bombax ceiba* Linn. in Wistar rats.

## Conclusion

The results obtained in present study may conclude that the extract of stem bark of *Bombax ceiba* Linn. has significant anti-obesity potential against HFD induced experimental obesity, possibly due to modulation of FAS and PTP-1B signaling in Wistar rats due to the presence of active flavanoids and lupeol respectively.

## Abbreviations

HFD: High fat diet; FAS: Fatty acid synthase; PTP-1B: Protein tyrosine phosphatase-1B; ALT: Alanine transferase; AST: Aspartate transferase.

## Competing interests

The authors declare that they have no competing interests.

## Authors’ contributions

RG and PS designed the experimental protocol. PG, YC and RG carried out the experimentation and drafted the manuscript. RG and PS performed the statistical analysis and interpreted the results. All authors read and approved the final manuscript.

## Pre-publication history

The pre-publication history for this paper can be accessed here:

http://www.biomedcentral.com/1472-6882/13/281/prepub

## References

[B1] Grill H Ginsberg A Seeley R Kaplan J Brainstem application of melanocortin receptor ligands produces long-lasting effects on feeding and body weight J Neurosci 1998 13 10128 10135 9822766 10.1523/JNEUROSCI.18-23-10128.1998 PMC6793290

[B2] Haslam DW James WP Obesity Lancet 2005 13 1197 1209 10.1016/S0140-6736(05)67483-1 16198769

[B3] Wilding JP Neuropeptides and appetite control Diabet Med 2001 13 619 627 10.1046/j.1464-5491.2002.00790.x 12147141

[B4] Zammit VA Waterman IJ Topping D McKay G Insulin stimulation of hepatic triacylglycerols secretion and the etiology of insulin resistance J Nutr 2001 13 2074 2077 11481396 10.1093/jn/131.8.2074

[B5] Klaman LD Boss O Peroni OD Kim JK Martino JL Zabolotny JM Moghal N Lubkin M Kim YB Sharpe AH Increased energy expenditure, decreased adiposity, and tissue-specic insulin sensitivity in protein-tyrosine phosphatase 1B-decient mice Mol Cell Biol 2000 13 5479 5489 10.1128/MCB.20.15.5479-5489.2000 10891488 PMC85999

[B6] Sharma H Chandola HM Ayurvedic concept of obesity, metabolic syndrome, and diabetes mellitus J Altern Complem Med 2011 13 549 552 10.1089/acm.2010.0690 21649521

[B7] Verma V Jalalpure SS Sahu A Bhardwaj LK Prakesh Y *Bombax ceiba* Linn: pharmacognostical, phytochemistry, ethnobotany, and pharmacology studies Int Pharm Sci 2011 13 62 68

[B8] Young-jae Y Nguyen-Hai N Young K Byung-Zun A Antiangiogenic activity of lupeol from *Bombax ceiba* stems bark Phyto Reas 2003 13 4 341 344 10.1002/ptr.1140 12722136

[B9] Saleem R Ahmad M Hussain SA Qazi AM Ahmad SI Hypotensive, hypoglycemic and toxicology studies on the flavonol C-glycoside shamimin from Bombax ceiba Planta Med 1999 13 331 334 10.1055/s-1999-14060 10364838

[B10] Dar A Faizi S Naqvi S Roome T Zikrur-Rehman S Ali M Firdous S Moin ST Analgesic and antioxidant activity of mangiferin and its derivatives: the structure activity relationship Biol Pharm Bull 2005 13 596 600 10.1248/bpb.28.596 15802793

[B11] Singh V Pandey RP Ethanobotany of Rajasthan India, scientific publishers Jodhpur 1998 13 58 59

[B12] Karnick CR Ethnobotanical records of drug plants described in valmiki Ramayana and their uses in the ayurvedic system of medicine Quart J Crude Drug Res 1975 13 143 154

[B13] Jain V Verma SK Katewa SS Anandjiwala S Sing B Free radical scavenging property of *Bombax ceiba* Linn. root Res J Med Plant 2011 13 462 470

[B14] Kumar S Evaluation of RBC membrane stabilization and antioxidant activity of *Bombax ceiba* in an in vitro method Int J Pharma and Bio Sci 2011 13 1

[B15] Chen J Zhuang D Cai W Xu L Li E Wu Y Sugiyama K Inhibitory effects of four plants flavonoids extracts on fatty acid synthase J Environ Sci 2009 13 S131 S134 10.1016/S1001-0742(09)60056-5 25084411

[B16] Hata K Hori K Murata J Takahashi S Remodeling of actin cytoskeleton in lupeol induced B16 2F2 cell differentiation J Biochem 2005 13 4 467 472 10.1093/jb/mvi151 16272141

[B17] Harborne JB Phytochemical methods 1998 London: Chapman and Hall 1 254 3

[B18] Botham PA Acute systemic toxicity prospects for tiered testing strategies Toxicol in Vitro 2004 13 227 230 10.1016/S0887-2333(03)00143-7 14757114

[B19] Shrinivasan K Viswanad B Asrat L Kaul CL Ramarao P Combination of high-fat-diet and low-dose steptozotocin-trated rat: a model for type 2 diabetes and pharmacological screening Pharmacol Res 2005 13 313 320 10.1016/j.phrs.2005.05.004 15979893

[B20] Novellie LB Dinij YS Galhardi CM Ebaid GMX Rodrigues HG Main F Fernandes AAH Cicogna AC Novellifilho JLVB Anthropometrical parameters and markers of obesity in rats Lab Animals 2007 13 111 119 10.1258/002367707779399518 17234057

[B21] Bernardis LL Prediction of carcass fat, waterand lean body mass from Lee’s nutritive ratio in rats with hypothalamic obesity Experientia 1970 13 789 790 10.1007/BF02232553 4914444

[B22] Ainsline DA Proietto J Fam BC Thormburn AW Short-term, high-fat diet low circulating leptin concentration in rats Am J Clin Nutr 2000 13 438 442 10648255 10.1093/ajcn/71.2.438

[B23] Trinder K Hiraga Y Nakamura N Kitajo A Linuma F Determination of glucose in blood using glucose oxidase-peroxidien Chem Phem Bulletin 1969 13 568 570

[B24] Allain CC Poon LS Chan CS Richmond W Fu PC Enzymatic determination of total serum cholesterol Clin Chem 1974 13 470 475 4818200

[B25] Friedewal WT Levy RI Fredrickson DS Estimation of the concentration of low-density lipoprotein in cholesterol in plasma, without use of the preparative ultracentrifuge Cin Chem 1972 13 499 502 4337382

[B26] Werner M Gabrielson DG Eastman J Ultramicro determination of serum triglyceride by bioluminescent assay Clin Chem 1981 13 268 271 7460277

[B27] Clin J IFCC method for the measurement of catalytic concentration of enzyme Chem Clin Biochem 1986 13 497

[B28] Christie WW Christie WW Preparation of ester derivatives of fatty acids for chromatographic analysis Advances in Lipid Methodology 1993 Dundee: Oily Press 69 111 2

[B29] Ohkawa Η Ohishi Ν Yagi K Assay for lipid peroxides in animal tissues by thiobarbituric acid reaction Anal Biochem 1979 13 351 10.1016/0003-2697(79)90738-3 36810

[B30] Ellman GL Tissue sulphydryl groups Arch Biochem Biophys 1959 13 70 77 10.1016/0003-9861(59)90090-6 13650640

[B31] Green LC Wagner DA Glogowski J Skipper PL Wishnok JS Tannenbaum SR Analysis of nitrate, nitrite, and nitrate in biological samples Anal Biochem 1982 13 131 138 10.1016/0003-2697(82)90118-X 7181105

[B32] Buettner R Parhofer KG Woenckhaus M Wrede CE Kunz-Schughart LA Scholmerich J Bollheimer LC Defining high fat diet rat models: metabolic and molecular effects of different fat types J Mol Endocrinol 2006 13 485 501 10.1677/jme.1.01909 16720718

[B33] Chen HC Farese RVJ Determination of adipocyte size by computer image analysis J Lipid Res 2002 13 986 989 12032175

[B34] Toruner F Akbay E Cakir N Sancak B Elbeg S Taneri F Aktürk M Karakoç A Ayva ZG Arslan M Effects of PPAR gamma and PPAR alpha agonists on serum leptin levels in diet-induced obese rats Horm Metab Res 2004 13 226 230 15114521 10.1055/s-2004-814452

[B35] Pang J Choi Y Park T Ilex paraguariensis exart ameliorate obesity induced by high fat diet: potential rale of AMPK in the visceral adipose tissue Arch Biochem Biophys 2008 13 2 178 185 10.1016/j.abb.2008.02.019 18314006

[B36] Woods SC Seeley RJ Rushing PA D’Alessio D Tso PA Controlled high fat diet induced an obese syndrome in rats J Nutr 2003 13 1081 1087 12672923 10.1093/jn/133.4.1081

[B37] Storlien LH Jenkins AB Chisholm DJ Pascoe WS Khouri S Kraegen EW Influence of dietary fat composition on development of insulin resistance in rats. Relationship to muscle triglyceride and n-3 fatty acids in muscle phospholipids Diabetes 1991 13 2 280 289 10.2337/diab.40.2.280 1991575

[B38] Gil-Campos M Canete R Gil A Hormones regulating lipid metabolism and plasma lipids in childhood obesity Int J Obes Relat Metab Disord 2003 13 S75 S80 10.1038/sj.ijo.0802806 15543224

[B39] Wanless IR Lentz JS Fatty liver hepatitis (steatohepatitis) and obesity: an autopsy study with analysis of risk factors Hepatology 1990 13 1106 1110 10.1002/hep.1840120505 2227807

[B40] Unger RH The physiology of cellular liporegulation Annu Rev Physiol 2003 13 333 347 10.1146/annurev.physiol.65.092101.142622 12471167

[B41] Hyashi T Hirshman MF Kurth EJ Winder WW Goodyear LJ Evidence for 5′AMP-activated protein kinase mediation of the effect of muscle contraction on glucose transport Diabetes 1998 13 1369 1373 9703344 10.2337/diab.47.8.1369

[B42] Day CP James OF Steatohepatitis: a tale of two hits Gastroenterology 1998 13 842 845 10.1016/S0016-5085(98)70599-2 9547102

